# Association of hospital and surgeon volume with mortality following major surgical procedures: Meta-analysis of meta-analyses of observational studies

**DOI:** 10.1097/MD.0000000000017712

**Published:** 2019-11-01

**Authors:** Hiroshi Hoshijima, Zen’ichiro Wajima, Hiroshi Nagasaka, Toshiya Shiga

**Affiliations:** a Department of Anesthesiology, Saitama Medical University Hospital, Saitama; b Department of Anesthesiology, Tokyo Medical University Hachioji Medical Center, Tokyo; c Department of Anesthesiology and Intensive Care Medicine, International University of Health and Welfare Ichikawa Hospital, Ichikawa, Chiba, Japan.

**Keywords:** caseload, centralization, hospital volume, mortality, surgeon volume

## Abstract

Accumulation of the literature has suggested an inverse association between healthcare provider volume and mortality for a wide variety of surgical procedures. This study aimed to perform meta-analysis of meta-analyses (umbrella review) of observational studies and to summarize existing evidence for associations of healthcare provider volume with mortality in major operations.

We searched MEDLINE, SCOPUS, and Cochrane Library, and screening of references.

Meta-analyses of observational studies examining the association of hospital and surgeon volume with mortality following major operations. The primary outcome is all-cause short-term morality after surgery. Meta-analyses of observational studies of hospital/surgeon volume and mortality were included. Overall level of evidence was classified as convincing (class I), highly suggestive (class II), suggestive (class III), weak (class IV), and non-significant (class V) based on the significance of the random-effects summary odds ratio (OR), number of cases, small-study effects, excess significance bias, prediction intervals, and heterogeneity.

Twenty meta-analyses including 4,520,720 patients were included, with 19 types of surgical procedures for hospital volume and 11 types of surgical procedures for surgeon volume. Nominally significant reductions were found in odds ratio in 82% to 84% of surgical procedures in both hospital and surgeon volume-mortality associations. To summarize the overall level of evidence, however, only one surgical procedure (pancreaticoduodenectomy) fulfilled the criteria of class I and II for both hospital and surgeon volume and mortality relationships, with a decrease in OR for hospital (0.42, 95% confidence interval[CI] [0.35–0.51]) and for surgeon (0.38, 95% CI [0.30–0.49]), respectively. In contrast, most of the procedures appeared to be weak or “non-significant.”

Only a very few surgical procedures such as pancreaticoduodenectomy appeared to have convincing evidence on the inverse surgeon volume-mortality associations, and yet most surgical procedures resulted in having weak or “non-significant” evidence. Therefore, healthcare professionals and policy makers might be required to steer their centralization policy more carefully unless more robust, higher-quality evidence emerges, particularly for procedures considered as having a weak or non-significant evidence level including total knee replacement, thyroidectomy, bariatric surgery, radical cystectomy, and rectal and colorectal cancer resections.

## Abstract 2

Supplemental Digital Content is available in the text

## 1 Introduction

Since its first introduction in the 1979 by Luft and colleagues,^[1]^ much literature has suggested an inverse association between healthcare provider volume and mortality for a wide variety of surgical procedures. Accumulation of supportive findings has been a major driving force towards a policy of “centralization”—selective referral from a low-volume hospital to a high-volume hospital. In the UK, Canada, and the Netherlands, programmed centralization has already been implemented for complex high-risk procedures.^[2–6]^ In the US, a national non-profit organization has advocated centralization by presenting minimum hospital and surgeon volume standards for 8 procedures.^[7]^

Centralization has made a great contribution to improved outcomes in complex surgical oncology represented by pancreatic resection.^[2,6]^ However, some criticisms still linger. First, there remains controversy over whether hospital/surgeon volume can be a precise measure of quality of care.^[8–11]^ Second, access to a high-volume hospital might be restricted especially for patients living in rural and underserved areas.^[11–14]^ Some experts express concern that such inaccessibility might aggravate the existing health disparities between patients with high and low socioeconomic status.^[2,11,15]^ Third, as operations are one of the crucial sources of income for hospitals, excessive centralization might plunge low-volume hospitals such as rural hospitals into financial difficulties, thereby causing serious consequences to local communities.^[11]^

The rationale for proponents of centralization might be based on “positive” results derived from observational studies and their meta-analyses. However, according to the GRADE (Grading of Recommendations, Assessment, Development and Evaluation) working group classification,^[16]^ the quality of evidence of those studies is considered “low” unless a large magnitude of effect, a dose-response gradient, or plausible confounding is certain.^[17]^ The quality of evidence in these studies has not been evaluated to date. Furthermore, these studies, and especially the meta-analyses, were limited to one particular procedure, and it remains uncertain which procedures have a strong volume-outcome relationship and which do not.

An umbrella reviews, which is performed to review existing systematic reviews and/or meta-analyses (meta-analysis of meta-analyses), provides nearly the highest level of evidence that can be presently obtained.^[18,19]^ The latest method of umbrella review provides a more comprehensive overview than other review methods do by using simultaneous assessment of *P* values, confidence intervals, prediction intervals, number of cases, largest study effects, heterogeneity, small-study effects, and excess significance bias.^[18]^ We, therefore, conducted an umbrella review of meta-analyses of observational studies to clarify whether healthcare provider volume might be associated with decreased mortality, and if so, to what extent, or whether it might depend on methodological quality, quality of evidence, or types of surgical procedures.

## 2 Methods

### 2.1 Umbrella review methods

Meta-analysis of meta-analyses (umbrella review) was conducted according to the practical guidance published by Aromataris et al^[18]^ and Fusar-Poli et al^[19]^ For reanalysis of each meta-analysis from the original cohort studies, we followed the reporting guidelines for Meta-analyses Of Observational Studies in Epidemiology (MOOSE) Statement.^[20]^ Ethical approval was not necessary because this study did not involve patient consent. The protocol for this umbrella review was registered in the University Hospital Medical Information Network in Japan (UMIN000033032).

### 2.2 Literature search

We searched MEDLINE, SCOPUS, and the Cochrane Library from inception through March 2018. We searched only meta-analyses that compared the mortality of patients who underwent various operations in a high-volume hospital versus a low-volume hospital or by a high-volume surgeon versus a low-volume surgeon. Each search strategy is detailed in Supplemental Content 1. Language restrictions were not applied. Unpublished studies and conference proceedings were excluded. A hand search of the references listed in eligible articles was also performed. All relevant titles and abstracts from the databases were imported into EndNote X8 (USACO Corporation, Tokyo, Japan) for further sorting. Two authors (HH, TS) independently screened the titles and abstracts. Disagreements were resolved by a third author (ZW).

### 2.3 Outcome measures and eligibility criteria

The primary outcome was defined as all-cause short-term mortality (30-day mortality or in-hospital mortality). The summary effect size was expressed as an odds ratio with corresponding 95% confidence interval (CI). The threshold of hospital/surgeon volume was defined according to the definition used in each original meta-analysis. Our inclusion criteria were as follows:

(1) the exposure is a “high-volume hospital” and/or “high-volume surgeon”;
(2) meta-analyses were conducted;
(3) dichotomous outcome measures (from forest plots) were available or could be calculated from the original cohort studies;
(4) effect sizes (e.g., odds ratio) with corresponding 95% CIs were available or could be derived from the original cohort studies; and
(5) sample size restrictions were not applied.

If more than one meta-analysis existed on the same surgical procedure, we included the latest meta-analysis; however, if more than one meta-analysis on the same type of operation was published in the same year, we finally included only one of them after consensus was obtained and compared them in the sensitivity analysis. Systematic reviews without meta-analytic methods were excluded because we were interested mainly in summary effects sizes rather than narrative opinions. We excluded meta-analyses whose authors did not present summary effect sizes with appropriate statistical methods and for which we could not reproduce the specific data from the original cohort studies they included. The meta-analyses focusing only on long-term mortality (often referred to as 1-year or 5-year survival rate) were also excluded.

### 2.4 Data extraction and synthesis

Data extraction was done in a two-level fashion to avoid using data resulting from the authors’ inappropriate statistical methods (e.g., only a fixed-effects model applied) or to correct insufficient data (e.g., absence of publication bias analysis). At the first level, we extracted information from each meta-analysis including the following data: type of operation, cases (deaths), population, number of studies included, name of the first author, year of publication, type of primary outcome, and cut-off threshold of high volume per year. If dichotomous data (e.g., a 2 × 2 contingency table) were available, we used this for further data synthesis. If not (e.g., odds ratio with corresponding 95% CI only), we moved onto the second level for which we obtained all of the primary study articles that the meta-analysis included and then extracted dichotomous data from them. If this succeeded, the data was synthesized; however, if it failed, data only on the effect size with 95% CIs were used for synthesis. If we failed to even collect data on effect size with 95% CIs, we excluded the meta-analysis from our umbrella review. Data extraction was performed independently by two investigators (HH, TS), and consensus was obtained with the third investigator (ZW) if there were disagreements.

### 2.5 Statistical analysis

We used both fixed and DerSimonian and Laird random-effects models^[21]^ to estimate the summary effect size (odds ratio) and the corresponding 95% CIs. We assessed the heterogeneity of effect size across studies using the Cochrane Q statistic and I^2^ statistic (I^2^ >60%: high heterogeneity; 40 to 60%: moderate heterogeneity; < 40%: low heterogeneity).

We estimated the 95% prediction intervals for the summary random effects odds ratio. The prediction interval provides information on how the true effects are distributed about the summary effect in a random-effects model.^[15]^ For instance, if 95% prediction intervals exceed zero, the true effect in 95% of the future studies will exclude the null value. A small-study effect (publication bias) was estimated by Egger regression test.^[22]^

We also used the excess significance test to estimate whether the observed number of studies (O) with statistically significant results (positive studies) was different from the expected number of positive studies (E).^[23]^ Briefly, we calculated E for each meta-analysis as the sum of the statistical power estimates for each individual study. The greater the disparity between O and E, the greater is the degree of excess significance bias.

A *P* value < .05 was considered significant for both the fixed- and random-effects odds ratios. A *P* value < .1 was considered significant for the excess significance test and Egger regression test. All the analyses were performed using STATA 15.0 (StataCorp, College Station, TX).

A sensitivity analyses was conducted when more than one meta-analysis on the same type of surgical procedures was published in the same year.

### 2.6 Stratification of evidence specific to an umbrella review

We performed an umbrella review-level stratification of evidence using modified criteria recommended by Fusar-Poli et al^[19]^:

- Convincing evidence (Class I) when the number of cases (deaths) > 1000, highly significant summary associations (random-effects *P* < 10^−6^), no evidence of small-study effects, no evidence of excess significance bias, 95% prediction intervals excluding the null, and not large heterogeneity (I^2^ < 50%);
- Highly suggestive evidence (Class II) when the number of cases > 1000, random-effects *P* < 10^−6^, and largest study with a statistically significant effect and class I criteria not met;
- Suggestive evidence (Class III) when the number of cases > 1000, random-effects *P* < 10^−3^, and class I-II criteria not met;
- Weak evidence (Class IV) when *P* < .05 and class I-III criteria not met or unclear; and
- Non-significant when *P* > .05.

### 2.7 Assessment of methodological quality and quality of evidence

We assessed the methodological quality of the meta-analyses by using AMSTAR 2 (A MeaSurement Tool to Assess systematic Reviews).^[24]^ AMSTAR 2 has adopted new evaluation system consisting of 16 items that evaluate 7 critical flaws and 9 non-critical weaknesses. Briefly, critical flaws include prior protocol registration, adequacy of the literature search, justification for excluding individual studies, risk of bias in individual studies, appropriateness of the meta-analytical methods, consideration of risk of bias, and assessment of publication bias. The final judgment by AMSTAR 2 in each meta-analysis can be categorized as “high,” “moderate,” “low,” or “critically low.”

We used the GRADE classification^[16]^ to assess the quality of evidence for mortality in each surgical procedure included in our umbrella review. Briefly, the GRADE system downgrades the quality of evidence when risk of bias, inconsistency, indirectness, or imprecision might be certain. Conversely, the GRADE system upgrades the quality of evidence when a large magnitude of effect, dose-response gradient, or a plausible confounder is present. The final judgment of GRADE in the outcome can be categorized as “high,” “moderate,” “low,” or “very low.” AMSTAR and GRADE were assessed independently by two investigators (HH, TS). Any differences between the two investigators were resolved by consensus.

### 2.8 Patient and public involvement

Patients were not involved in determining research questions or outcome measures or in designing or implementing the present study. The patients were not asked for their opinions on interpreting or writing the results. The results of the present study will not be disseminated to the study participants or other relevant parties.

## 3 Results

### 3.1 Study selection and characteristics

**Figure 1:**
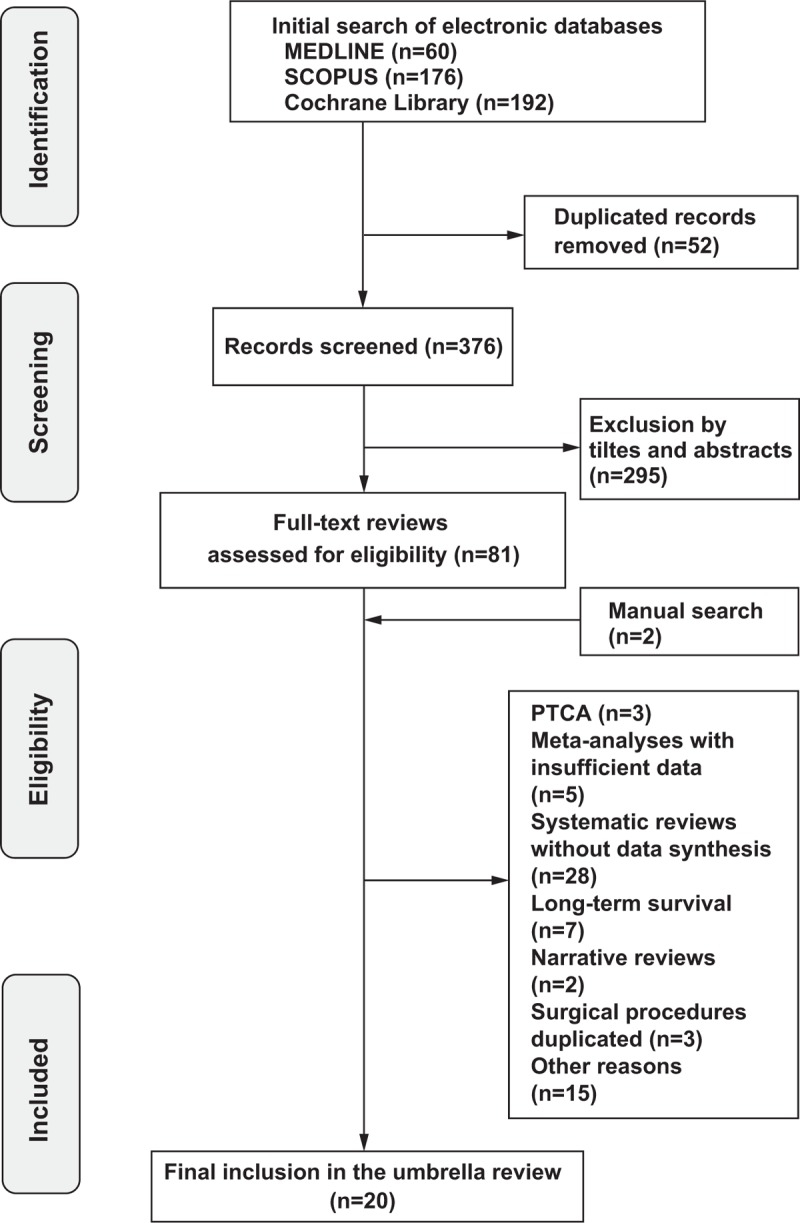
PRISMA flow diagram for literature search, study screening and selection.

We finally included 20 meta-analyses^[25–44]^ with a total of 4,520,720 patients after the systematic search and selection of eligible reviews (see Fig. 1). Nineteen were written in English, and one was written in German.^[41]^ The search yield 26 types of surgical procedures for both hospital and surgeon volume and mortality associations (19 for hospital volume and 11 for surgeon volume). The literature excluded from the full-text reviews and the reasons for doing so are listed in Supplemental content 2. The characteristics of the extracted data, calculated summary effect sizes, heterogeneity, publication bias, and excess significance are tabulated in Tables 1 and 2.

**Table 1:**
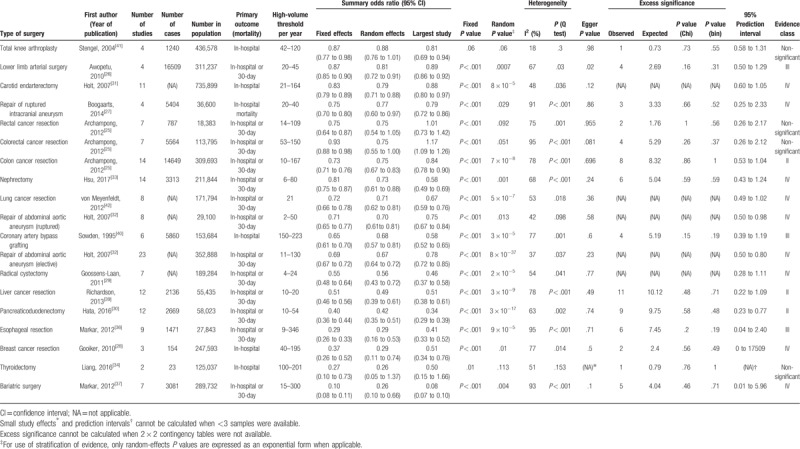
Summary of 19 meta-analyses on the association between hospital volume and mortality in the umbrella review.

**Table 2:**
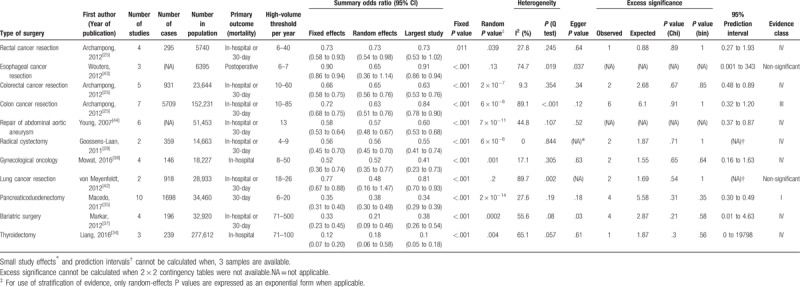
Summary of 11 meta-analyses on the association between surgeon volume and mortality in the umbrella review.

### 3.2 Summary effect size

**Figure 2:**
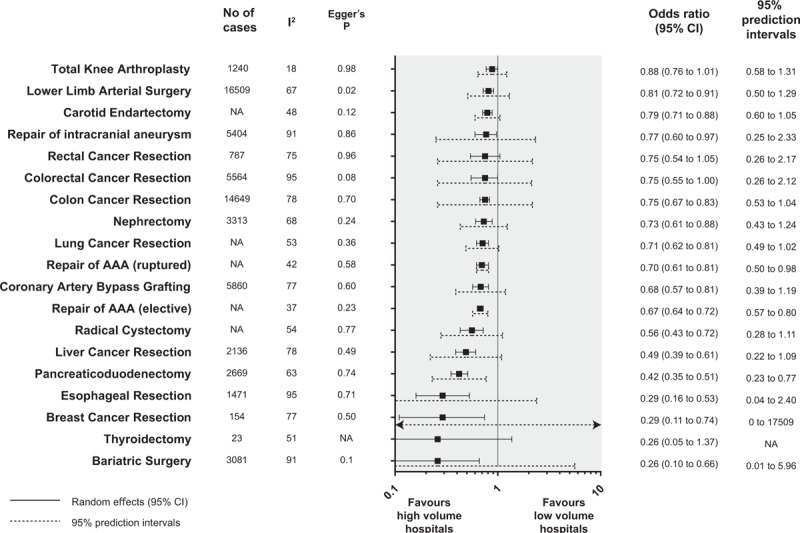
Summary random effects estimates with 95% confidence and prediction intervals from 19 meta-analyses on the association between hospital volume and mortality. AAA = abdominal aortic aneurysm; NA = not applicable.

**Figure 3:**
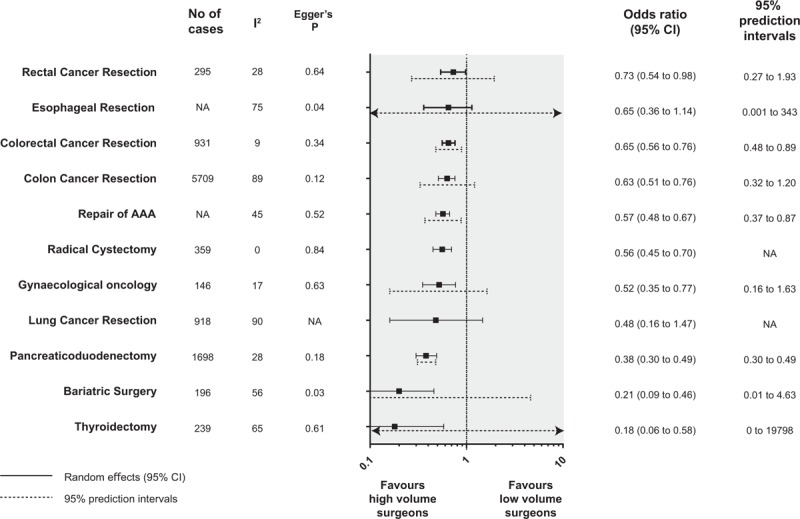
Summary random effects estimates with 95% confidence and prediction intervals from 11 meta-analyses on the association between hospital volume and mortality. AAA = abdominal aortic aneurysm; NA = not applicable.

For hospital volume and mortality associations, the summary random effects estimates were significant (*P* < .05) in 15 of 19 surgical procedures (79%), whereas the summary fixed effect estimates were significant in all surgical procedures (100%) (see Figs. 2 and 3). In 15 surgical procedures (84%), the effects of the largest study were significant. Regarding estimation of 95% prediction intervals, the null value was excluded in only 3 surgical procedures (repair of abdominal aortic aneurysm^[32]^ [both elective and ruptured], and pancreaticoduodenectomy^[30]^).

For surgeon volume and mortality associations, the summary random effects estimates were significant in 9 of 11 surgical procedures (82%), whereas the summary fixed effects estimates were significant in all surgical procedures (100%). The effects of the largest study were significant in 10 surgical procedures (91%). Regarding estimation of 95% prediction intervals, the null value was excluded in only three surgical procedures (repair of abdominal aortic aneurysm,^[44]^ colorectal cancer,^[25]^ and pancreaticoduodectomy^[35]^).

### 3.3 Heterogeneity among studies

For hospital volume and mortality associations, significant heterogeneity (*P* < .10) was observed in 17 of 19 surgical procedures (89%). High heterogeneity (I^2^ > 60) was identified in 12 surgical procedures (63%), moderate heterogeneity (I^2^ = 40 to 60) in 5 surgical procedures (26%), and low heterogeneity (I^2^ < 40) in 2 surgical procedures (11%).

For surgeon volume and mortality associations, significant heterogeneity was detected in 6 of 11 surgical procedures (55%). High heterogeneity (I^2^ > 60) was identified in 4 surgical procedures (36%), moderate heterogeneity (I^2^ = 40 to 60) in 2 surgical procedures (18%), and low heterogeneity (I^2^ < 40) in 5 surgical procedures (45%).

### 3.4 Small-study effects

Small-study effects could not be calculated in one and one surgical procedure in the hospital and surgeon volume and mortality relations, respectively, due to an inadequate number of studies. For hospital volume and mortality associations, a small-study effect, as assessed using Egger test, was observed in 2 of 18 (one procedure was not applicable due to the small numbers of studies included) surgical procedures (11%). For surgeon volume and mortality associations, a small-study effect was detected in 2 of 10 (one was not applicable) surgical procedures (20%).

### 3.5 Excess significance

Excess significance could not be calculated in 5 and 2 surgical procedures for hospital and surgeon volume and mortality relations, respectively, because 2 × 2 contingency tables were not available. For the rest of the procedures, there was no evidence of excess significance bias for each surgical procedure in either hospital or surgeon volume and mortality associations. For hospital volume and mortality associations, among all 162 individual studies included, the O value was 66 whereas the E value was 66.9. For surgeon volume and mortality associations, among all 50 individual studies included, O was 24 whereas E was 25.1.

### 3.6 Stratification of evidence specific to umbrella reviews

For hospital volume and mortality associations, no surgical procedures were classified as “class I,” indicating that convincing evidence was absent. Three procedures (16%) (pancreaticoduedectomy,^[30]^ liver cancer resection,^[39]^ and colon cancer resection^[25]^) were categorized as “class II (highly suggestive).” Another three procedures (16%) were categorized as “class III (suggestive),” nine procedures (47%) as “class IV (weak),” and four procedures (21%) as “non-significant.”

For surgeon volume and mortality associations, convincing evidence (class I) was identified in one surgical procedure (9%) (pancreaticoduodenectomy^[35]^). No procedures were categorized as “class II”. One procedure (colon cancer resection) (9%) was categorized as “class III,” 7 procedures (64%) as “class IV,” and 2 procedures (18%) as “non-significant.”

### 3.7 AMSTAR 2 and GRADE classification

**Figure 4:**
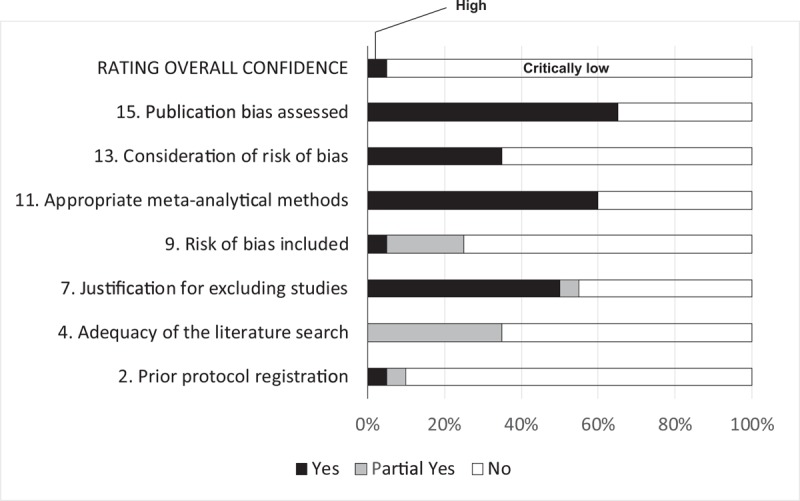
Results of AMSTAR 2 assessment (n = 20 meta-analyses). Among 16 items, only 7 critical domains and overall rating were indicated (see also supplemental Table 1).

Figure 4 shows an overall summary of the AMSTAR 2 rating across the 20 meta-analyses. The rating of overall confidence in 1 meta-analysis^[25]^ was judged as “high,” whereas that in the rest of the meta-analyses was judged as “critically low.” Detailed information on the results of AMSTAR 2 are shown in Supplemental Content 3. Specifically, in item 2, Prior protocol registration, only 2 meta-analyses^[25,38]^ (10%) had evidence of registration being accomplished (e.g., Cochrane Database of Systematic Reviews^[25]^ or PROSPERO^[38]^), but we could not find any information on a prespecified protocol or registration for any of the other meta-analyses. In item 4, Adequacy of the literature search, 6 meta-analyses^[26,30,33,35,42,43]^ (30%) restricted the language to English, although no justification for this was provided, and it was unclear in seven other meta-analyses^[29,31,32,36,39,40,44]^ (35%) whether a language restriction was applied at all. In item 11, Appropriateness of meta-analytical methods, two meta-analyses^[31,44]^ (10%) reported the use of a fixed-effects model only. In item 16, Reporting of any potential sources of conflict of interest, including any funding the authors received for conducting the review, six meta-analyses^[31,32,35,36,39,44]^ (30%) did not report either no competing interests or their funding sources.

For hospital volume and mortality associations, the final judgment of GRADE categorized two surgical procedures^[32,41]^ (11%) as “low” and 17 surgical procedures (90%) as “very low.” For surgeon volume and mortality associations, the final judgment of GRADE categorized four surgical procedures^[25,29,35]^ (36%) as “low” and seven surgical procedures (64%) as “very low.” Supplemental Content 4 shows the GRADE evidence profile representing the certainty assessment and the GRADE scores for mortality in each surgical procedure.

### 3.8 Sensitivity analysis

Two meta-analyses^[36,43]^ on hospital volume and mortality association in esophageal resection were published in the same year (2012) (see Table, Supplemental Content 5). Dichotomous data were available in one meta-analysis^[36]^ but not in the other^[43]^; therefore, we finally included the former meta-analysis in our umbrella review. However, the latter meta-analysis was also included in the analysis of surgeon volume and mortality association because the odds ratio and 95% CI were available. Comparison of the 2 meta-analyses is shown in supplemental Table 3. Both meta-analyses were notably different in the number of studies included, publication bias, and 95% prediction interval. The methodological quality (AMSTAR 2) and quality of evidence (GRADE) were the same in these 2 meta-analyses (“critically low” and “very low,” respectively).

## 4 Discussion

We found nominally significant reductions in the random-effects odds ratio in 84% of the surgical procedures in the hospital volume and mortality associations, and in 82% of the surgical procedures in the surgeon volume and morality associations. Nevertheless, the prediction intervals excluded the value of 1.0 in a few surgical procedures in both the hospital and surgeon volume relationships. This means that the true odds ratio in 95% of the future studies will not exceed the value of 1.0 for most of the surgical procedures. A low degree of heterogeneity was observed in several surgical procedures, whilst small-study effects were not observed in most of the surgical procedures, and excess significance bias was not found in any of the surgical procedures.

Summarizing the above in the context of an umbrella review-level stratification of evidence, only one surgical procedure—pancreaticoduodenectomy—fulfilled the criteria of convincing (class I) and highly suggestive (class II) evidence in both the hospital and surgeon volume and mortality relationships. That is, it is certain that pancreaticoduodenectomy performed in high-volume hospitals or by high-volume surgeons reduced all-cause short-term mortality by 58% or 62%, respectively. Strong correlations were found, and this result is in accordance with the common understanding that centralization has improved mortality in pancreaticoduodenectomy, which is representative of a surgical procedure of the highest complexity. In contrast, most of the evidence for the surgical procedures in the hospital volume- and surgeon volume-mortality relationship appeared to be weak (class IV) or “non-significant,” indicating that robust evidence on the association of healthcare provider volume and mortality was sparse in the currently available meta-analyses.

However, robust evidence is valid only when methodological flaws do not exist in each meta-analysis. Our assessment by AMSTAR 2 shows that only one meta-analysis, that registered with the Cochrane center,^[25]^ resulted in a high rating, whereas all of the other meta-analyses were rated as “critically low.” Even pancreaticoduodenectomy could not escape from inherent methodological flaws. Notably, most of the meta-analyses did not accomplish prespecified protocol registration, implying that they are vulnerable to selective inclusion and reporting. Only 7 meta-analyses were free from language restriction. More critically, it was unclear whether language restriction was even applied at all in another 7 meta-analyses. Bias can be easily introduced when a meta-analysis is exclusively based on English-language papers alone.^[45]^

Furthermore, the quality of evidence as assessed by GRADE was rated as “very low” in most of the meta-analyses, and only a few were rated as “low.” A randomized controlled trial is difficult to perform for this type of the research question, probably due to ethical considerations; thus, results from observational studies may be the best evidence available at present and in the future. Basically, observational studies are categorized as “low.” A large magnitude of effect, a dose-response gradient, or plausible confounding is a prerequisite for upgrading to “high.” The meta-analyses on pacreaticoduedecotomy^[30,35]^ could have been upgraded by strong associations (odds ratio < 0.5), but actually, they were downgraded by other factors including heterogeneity or absence of risk of bias assessment.

Our sensitivity analysis showed that the evidence level for esophageal resection in our umbrella review was “suggestive” for a hospital volume and mortality relationship.^[36]^ Since Birkmeyer et al^[46]^ published their paper in the early 2000s, the results of improved outcomes in esophageal resection have played a major role in pushing forward for centralization. Nevertheless, our results were quite disappointing. Furthermore, two similar meta-analyses^[36,43]^ were published in the same year. Substantial inconsistency was present between these 2 meta-analyses with respect to heterogeneity, publication bias, and prediction interval, whilst the magnitude of the odds ratio and the AMSTAR 2 and GRADE classifications were similar. The plausible explanation for this is that each meta-analysis chose different studies. One included 9 studies,^[36]^ whereas the other included 16 studies,^[43]^ and more surprisingly, no studies overlapped despite the selection of similar databases and similar search periods. In any case, 6 years have passed since both were published, and an updated meta-analysis on esophageal resection is needed soon.

The strengths of our umbrella review can be appreciated from a comparison with three previously published systematic reviews of systematic reviews without meta-analytic approaches.^[47–49]^ The strengths of our umbrella review can be appreciated from a comparison with 3 previously published systematic reviews of systematic reviews performed without applying meta-analytic approaches. Although all 3 reviews dealt with a wide variety of operations including percutaneous coronary intervention and mixed short-term and long-term outcomes were presented, the strength of our umbrella review lies in its conduction according to practical guidelines, with risk of bias and GRADE assessed with quantitative evaluations of prediction interval, excess significance, and other factors. Our study has several limitations. First, the definition of high-volume threshold varies from study to study. This might result in substantial heterogeneity in many of the meta-analyses included. It is a potential disadvantage to use provider volume as a quality indicator in this kind of study addressing the theme of volume-outcome relationships. Second, the meta-analyses included in our review spanned two decades (from 1995 to 2017) during which advancements in surgical techniques might have improved outcomes; therefore, caution is advised when discussing these meta-analyses together. Specifically, the meta-analyses published before 2010 need to be updated.

Which factor is more relevant to improving mortality, a high-volume hospital or a high-volume surgeon? This question may be more complicated by the paradox often mentioned of how do we interpret a situation in which a high-volume hospital uses low-volume surgeons or a high-volume surgeon practices in a low-volume hospital? The perception for our review is that the level of evidence for the relationship between a high-volume hospital and mortality ranked higher than that between a high-volume surgeon and mortality: however, which factor might most affect patient outcomes remains unclear. A future work using a multi-level approach (patient level, surgeon level, and hospital level) may shed some light on this question by, for instance, using a generalized linear mixed model to clarify how interactively and to what extent each factor affects an improvement in outcomes.

Policy makers and insurance companies should not expand the indications for centralization until higher-quality, more convincing evidence emerges, particularly for procedures that appeared to have a weak or non-significant evidence level such as total knee replacement, thyroidectomy, bariatric surgery, radical cystectomy, and rectal and colorectal cancer resections. However, policy makers also need to continue centralization for more complex surgical procedures such as pancreaticoduodenectomy, within a range that does cause unwanted secondary effects.

In conclusion, although healthcare provider volume and mortality have been extensively investigated over the past three decades, only a very few surgical procedures such as pancreaticoduodenectomy appear to have convincing evidence for an inverse surgeon volume-mortality relationship, and yet most surgical procedures resulted in having weak or “non-significant” evidence. Therefore, healthcare professionals and policy makers might be required to steer their centralization policy more carefully unless more robust, higher-quality evidence emerges, particularly for procedures considered as having a weak or non-significant evidence level, including total knee replacement, thyroidectomy, bariatric surgery, radical cystectomy, and rectal and colorectal cancer resections.

## Acknowledgments

We thank Toshiro Tango, PhD (Center for Medical Statistics, Tokyo, Japan) for statistical consulting. We also thank George B. Powell of the firm Rise Japan for editing the manuscript.

## Author contributions

**Conceptualization:** Toshiya Shiga.

**Data curation:** Hiroshi Hoshijima, Zen’ichiro Wajima, Toshiya Shiga.

**Formal analysis:** Hiroshi Hoshijima, Zen’ichiro Wajima, Toshiya Shiga.

**Funding acquisition:** Toshiya Shiga.

**Software:** Toshiya Shiga.

**Supervision:** Zen’ichiro Wajima, Hiroshi Nagasaka, Toshiya Shiga.

**Validation:** Hiroshi Hoshijima, Zen’ichiro Wajima.

**Writing – original draft:** Toshiya Shiga.

**Writing – review & editing:** Hiroshi Hoshijima, Zen’ichiro Wajima, Hiroshi Nagasaka, Toshiya Shiga.

## Supplementary Material

Supplemental Digital Content
