# Supplementary material for: Association of hospital and surgeon volume with mortality following major surgical procedures: Meta-analysis of meta-analyses of observational studies
Source: Medicine (Baltimore). 2019 Nov 1;98(44):e17712. doi: 10.1097/MD.0000000000017712 (PMC6946306; doi:10.1097/MD.0000000000017712)
Supplement: Supplemental Digital Content [file medi-98-e17712-s001.pdf]

Supplemental Conent 1

MEDLINE search strategy

("hospital volume"[All Fields] OR "surgeon volume"[All Fields] OR "surgical volume"[All Fields] OR ("workload"[MeSH Terms] OR "workload"[All Fields]) OR caseload[All Fields] OR "procedure volume"[All Fields] OR "procedural volume"[All Fields]) AND (("mortality"[Subheading] OR "mortality"[All Fields] OR "mortality"[MeSH Terms]) OR ("mortality"[Subheading] OR "mortality"[All Fields] OR "survival"[All Fields] OR "survival"[MeSH Terms]) OR "treatment outcome"[All Fields]) AND "meta-analysis"[publication type])

Scopus search strategy

TITLE-ABS-KEY({hospital volume} OR {surgeon volume} OR {surgical volume} OR workload OR caseload OR {procedure volume} OR {procedural volume}) AND TITLE-ABS-KEY(mortality OR survival) AND TITLE-ABS-KEY("meta-analysis" OR "systematic review")

Cochrane library search strategy

("hospital volume" or "surgeon volume" or "surgical volume" or workload or caseload or "procedure volume" or "procedural volume") and (mortality or survival or "treatment outcome") and ("meta-analysis" or "systematic review")

Supplemental Content 2. The list of excluded full-text articles with the reasons for exclusion

Duplicated surgical procedures

1. Gooiker GA, Van Gijn W, Wouters MW, et al. Systematic review and meta-analysis of the volume-outcome relationship in pancreatic surgery. Br J Surg 2011;98:485-94.
2. Mayer EK, Purkayastha S, Athanasiou T, et al. Assessing the quality of the volume-outcome relationship in uro-oncology. BJU Int 2009;103:341-9.
3. van Gijn W, Gooiker GA, Wouters MW, et al. Volume and outcome in colorectal cancer surgery. Eur J Surg Oncol 2010;36 Suppl 1:S55-63.

Percutaneous coronary intervention

1. Lin X, Tao H, Cai M, et al. A Systematic review and meta-Analysis of the relationship between hospital volume and the outcomes of percutaneous coronary intervention. Medicine (Baltimore) 2016;95:e2687.
2. Post PN, Kuijpers M, Ebels T, et al. The relation between volume and outcome of coronary interventions: a systematic review and meta-analysis. Eur Heart J 2010;31:1985-92.
3. Strom JB, Wimmer NJ, Wasfy JH, et al. Association between operator procedure volume and patient outcomes in percutaneous coronary intervention: a systematic review and meta-analysis. Circ Cardiovasc Qual Outcomes 2014;7:560-6.

Meta-analyses with insufficient information

1. Gruen RL, Pitt V, Green S, et al. The effect of provider case volume on cancer mortality: systematic review and meta-analysis. CA Cancer J Clin 2009;59:192-211.
2. Kalant N, Shrier I. Volume and outcome of coronary artery bypass graft surgery: Are more and less the same? Can J Cardiol 2004;20:81-86.
3. Langenhuijsen J, Birtle A, Klatte T, et al. Surgical management of adrenocortical carcinoma:

Impact of laparoscopic approach, lymphadenectomy, and surgical volume on outcomes-A systematic review and meta-analysis of the current literature. Eur Urol Focus 2016;1:241-50.

4. Saratzis A, Nduwayo S, Bath MF, et al. Impact of hospital volume on outcomes following treatment of thoracic aortic aneurysms and type-B dissections. Interact Cardiovasc Thorac Surg 2016;23:477-85.

5. Wilt TJ, Shamliyan TA, Taylor BC, et al. Association between hospital and surgeon radical prostatectomy volume and patient outcomes: A systematic review. J Urol 2008;180:820-29.

Systematic reviews without meta-analyses

1. Amato L, Colais P, Davoli M, et al. [Volume and health outcomes: evidence from systematic reviews and from evaluation of Italian hospital data]. Epidemiol Prev 2013;37(2-3 Suppl 2):1-100.

2. Bollschweiler E, Metzger R, Vallböhmer D, et al. Minimum case loads in visceral surgery - What is crucial: The surgical center or the single surgeon? Chir Gastroenterol 2008;24:274-79.

3. Caputo LM, Salottolo KM, Slone DS, et al. The relationship between patient volume and mortality in American trauma centres: A systematic review of the evidence. Injury 2014;45:478-86.

4. Chowdhury MM, Dagash H, Pierro A. A systematic review of the impact of volume of surgery and specialization on patient outcome. Br J Surg 2007;94:145-61.

5. Critchley RJ, Baker PN, Deehan DJ. Does surgical volume affect outcome after primary and revision knee arthroplasty? A systematic review of the literature. Knee 2012;19:513-18.

6. Davoli M, Amato L, Minozzi S, et al. Volume and health outcomes: an overview of systematic reviews. Epidemiol Prev 2005;29(3-4 Suppl):3-63.

7. Dikken JL, Stiekema J, Van De Velde CJ, et al. Quality of care indicators for the surgical treatment of gastric cancer: A systematic review. Ann Surg Oncol 2013;20:381-98.

8. Gandjour A, Bannenberg A, Lauterbach KW. Threshold volumes associated with higher

survival in health care: A systematic review. Med Care 2003;41:1129-41.

9. Henebiens M, van den Broek TA, Vahl AC, et al. Relation between hospital volume and outcome of elective surgery for abdominal aortic aneurysm: A systematic review. Eur J Vasc Endovasc Surg 2007;33:285-92.

10. Koy T, König DP, Eysel P. [Effects of hospital and surgeon procedure volume on outcome in total hip replacement]. Z Orthop Unfall 2007;145:291-6.

11. Lau RL, Perruccio AV, Gandhi R, et al. The role of surgeon volume on patient outcome in total knee arthroplasty: A systematic review of the literature. BMC Musculoskelet Disord 2012;13

12. Lauder CI, Marlow NE, Maddern GJ, et al. Systematic review of the impact of volume of oesophagectomy on patient outcome. ANZ J Surg 2010;80:317-23.

13. Marlow NE, Barraclough B, Collier NA, et al. Effect of hospital and surgeon volume on patient outcomes following treatment of abdominal aortic aneurysms: A systematic review. Eur J Vasc Endovasc Surg 2010;40:572-79+e57+e63.

14. Marlow NE, Barraclough B, Collier NA, et al. Centralization and the relationship between volume and outcome in knee arthroplasty procedures. ANZ J Surg 2010;80:234-41.

15. McAteer JP, LaRiviere CA, Drugas GT, et al. Influence of surgeon experience, hospital volume, and specialty designation on outcomes in pediatric surgery. JAMA Pediatr 2013;167:468-75.

16. Nuttall M, van der Meulen J, Phillips N, et al. A systematic review and critique of the literature relating hospital or surgeon volume to health outcomes for 3 urological cancer procedures. J Urol 2004;172(6 I):2145-52. doi: 10.1097/01.ju.0000140257.05714.45

17. Padwal R, Klarenbach S, Wiebe N, et al. Bariatric surgery: A systematic review of the clinical and economic evidence. J Gen Intern Med 2011;26:1183-94.

18. Pieper D, Mathes T, Asfour B. A systematic review of the impact of volume of surgery and specialization in Norwood procedure. BMC Pediatr 2014;14

19. Salz T, Sandler RS. The Effect of hospital and surgeon volume on outcomes for rectal cancer surgery. Clin Gastroenterol Hepatol 2008;6:1185-93.

20. Sepehripour AH, Athanasiou T. Is there a surgeon or hospital volume-outcome relationship in off-pump coronary artery bypass surgery? Interact Cardiovasc Thorac Surg 2013;16:202-07.

21. Singh JA, Kundukulam J, Riddle DL, et al. Early postoperative mortality following joint arthroplasty: A systematic review. J Rheumatol 2011;38:1507-13.

22. Slim K, Flamein R, Chipponi J. Relationship between surgeon's volume and outcomes: Myth or reality? Ann Chir 2002;127:502-11.

23. Thompson M, Holt P, Loftus I, et al. Debate: Whether abdominal aortic aneurysm surgery should be centralized at higher-volume centers. J Vasc Surg 2011;54:1208-14.

24. van Heek NT, Kuhlmann KF, Scholten RJ, et al. Hospital volume and mortality after pancreatic resection: A systematic review and an evaluation of intervention in The Netherlands. Ann Surg 2005;242:781-90.

25. Tol JA, van Gulik TM, Busch OR, et al. Centralization of highly complex low-volume procedures in upper gastrointestinal surgery. A summary of systematic reviews and meta-analyses. Dig Surg 2012;29:374-83.

26. Trinh QD, Bjartell A, Freedland SJ, et al. A systematic review of the volume-outcome relationship for radical prostatectomy. Eur Urol 2013;64:786-98. doi: 10.1016/j.eururo.2013.04.012

27. Wilson A, Marlow NE, Maddern GJ, et al. Radical prostatectomy: A systematic review of the impact of hospital and surgeon volume on patient outcome. ANZ J Surg 2010;80(1-2):24-29.

28. Zevin B, Aggarwal R, Grantcharov TP. Volume-outcome association in bariatric surgery: A systematic review. Ann Surg 2012;256:60-71.

Reviews assessing long-term survival only

1. Ahmed S, Luks FI, O'Brien BM, et al. Influence of experience, case load, and stage

distribution on outcome of endoscopic laser surgery for TTTS: A review. Prenat Diagn 2010;30:314-19.

2. Barocas DA, Cookson MS. Surgical management of clinically localized prostate cancer. Urol Oncol2010:255-66.
3. Brusselaers N, Mattsson F, Lagergren J. Hospital and surgeon volume in relation to long-term survival after oesophagectomy: Systematic review and meta-analysis. Gut 2014;63:1393-400.
4. du Bois A, Rochon J, Pfisterer J, et al. Variations in institutional infrastructure, physician specialization and experience, and outcome in ovarian cancer: A systematic review. Gynecol Oncol 2009;112:422-36.
5. Eskander A, Merdad M, Irish JC, et al. Volume-outcome associations in head and neck cancer treatment: a systematic review and meta-analysis. Head Neck 2014;36:1820-34.
6. Iversen LH, Harling H, Laurberg S, et al. Influence of caseload and surgical speciality on outcome following surgery for colorectal cancer: A review of evidence. Part 2: Long-term outcome. Colorectal Dis 2007;9:38-46.
7. Woo YL, Kyrgiou M, Bryant A, et al. Centralisation of services for gynaecological cancer. Cochrane Database Syst Rev 2012; . http://onlinelibrary.wiley.com/doi/10.1002/14651858.CD007945.pub2/abstract.

Narrative reviews

1. Heil J, Rauch G, Szabo AZ, et al. Breast cancer mastectomy trends between 2006 and 2010: association with magnetic resonance imaging, immediate breast reconstruction, and hospital volume. Ann Surg Oncol 2013;20:3839-46.
2. Karthikesalingam A, Hinchliffe RJ, Loftus IM, et al. Volume-outcome relationships in vascular surgery: The current status. J Endovasc Ther 2010;17:356-65.

Other reasons

1. Increased hospital and physician volumes associated with increased survival. Evidence-Based Healthcare and Public Health 2004;8:255-56.
2. Ahmed N, Devitt KS, Keshet I, et al. A systematic review of the effects of resident duty hour restrictions in surgery: impact on resident wellness, training, and patient outcomes. Ann Surg 2014;259:1041-53.
3. Baldwin K, Namdari S, Donegan D, et al. Early effects of resident work-hour restrictions on patient safety: A systematic review and plea for improved studies. J Bone Joint Surg Am 2011;93:e5.1-e5.9.
4. Brand CA, Barker AL, Morello RT, et al. A review of hospital characteristics associated with improved performance. Int J Qual Health Care 2012;24:483-94.
5. Davies RJ. A systematic review of the impact of volume of surgery and specialization on patient outcome (Br J Surg 2007; 94: 145-161) [7]. Br J Surg 2007;94:645.
6. Engineer LD, Winters BD, Weston CM, et al. Hospital characteristics and the agency for healthcare research and quality inpatient quality indicators: A systematic review. J Healthc Qual 2016;38:304-13.
7. Forbes TL, Ricco JB. Editors' commentary. J Vasc Surg 2011;54:1214.
8. Hastan D, Vandenbroucke JP, van der Mey AG. A meta-analysis of surgical treatment for vestibular schwannoma: is hospital volume related to preservation of facial function? Otol Neurotol 2009;30:975-80.
9. Hoornweg LL, Storm-Versloot MN, Ubbink DT, et al. Meta analysis on mortality of ruptured abdominal aortic aneurysms. Eur J Vasc Endovasc Surg 2008;35:558-70.
10. Koelemay MJ, Vahl AC. Meta-analysis and systematic review of the relationship between volume and outcome in abdominal aortic aneurysm surgery [1]. Br J Surg 2007;94:1041.
11. Luft HS, Parker JD. Volume and mortality in coronary artery bypass grafting. BMJ 1995;311:1304.
12. Mastracci TM, Garrido-Olivares L, Cinà CS, et al. Endovascular repair of ruptured abdominal

aortic aneurysms: a systematic review and meta-analysis. J Vasc Surg 2008;47:214-21.

13. Paterson-Brown S. Surgical volume and clinical outcome. Br J Surg 2007;94:523-24.

14. Pennathur A, Luketich JD. Resection for esophageal cancer: Strategies for optimal management. Ann Thorac Surg 2008;85:S751-S56.

15. Wilson TG, Guru K, Rosen RC, et al. Best practices in robot-assisted radical cystectomy and urinary reconstruction: Recommendations of the Pasadena Consensus Panel. Eur Urol 2015;67:363-75.

Supplemental Content 3. Results of AMSTAR 2 assessment

Author	Publication year	Items																Rating of overall confidence in the results of the review
		1	2	3	4	5	6	7	8	9	10	11	12	13	14	15	16	
von Meyenfeldt[43]	2012	Yes	No	Yes	No	Yes	Yes	Partial Yes	Yes	No	No	Yes	No	Yes	Yes	Yes	Yes	Critically low
Liang[35]	2016	Yes	No	Yes	Partial Yes	Yes	No	Yes	Yes	No	No	Yes	No	No	Yes	No	Yes	Critically low
Boogaarts[28]	2014	Yes	No	Yes	Partial Yes	Yes	Yes	Yes	Yes	No	No	No	No	No	No	Yes	No	Critically low
Markar[38] (Bariatric surgery)	2012	Yes	No	Yes	No	Yes	No	No	Yes	No	No	Yes	No	No	No	No	Yes	Critically low
Markar[36] (Oesophageal)	2012	Yes	No	Yes	No	Yes	No	Yes	Yes	No	No	Yes	No	No	Yes	Yes	No	Critically low
Hata[31]	2016	Yes	No	Yes	No	Yes	Yes	No	Yes	No	No	No	No	No	Yes	Yes	No	Critically low
Gooiker[29]	2011	Yes	No	Yes	Partial Yes	Yes	Yes	No	Yes	No	No	Yes	No	Yes	Yes	Yes	Yes	Critically low
Goossens-Laan[30]	2011	Yes	No	Yes	No	Yes	No	No	Yes	No	No	Yes	Yes	Yes	Yes	Yes	Yes	Critically low
Holt[33] (AAA)	2007	Yes	No	Yes	No	No	Yes	No	Partial Yes	No	No	No	No	No	No	No	No	Critically low
Richardson[40]	2013	Yes	No	Yes	Partial Yes	Yes	Yes	No	Yes	No	No	Yes	No	No	No	Yes	No	Critically low
Awopetu[27]	2010	Yes	No	Yes	No	No	Yes	Yes	Yes	No	No	No	No	No	Yes	Yes	Yes	Critically low
Holt[32] (Carotid)	2007	Yes	No	Yes	No	No	No	Yes	No	No	No	No	No	No	No	Yes	No	Critically low
Sowden[41]	1995	Yes	No	Yes	No	No	No	Yes	Yes	No	No	Yes	No	No	Yes	No	Yes	Critically low
Archampong[26]	2012	Yes	Yes	Yes	Partial Yes	Yes	Yes	Yes	Yes	Yes	No	Yes	Yes	Yes	Yes	Yes	Yes	High
Hsu[34]	2017	Yes	No	Yes	No	Yes	Yes	No	Yes	Partial Yes	No	No	No	No	Yes	Yes	Yes	Critically low
Mowat[39]	2016	Yes	Partial Yes	Yes	Partial Yes	No	Yes	Yes	Yes	No	No	Yes	Yes	Yes	Yes	No	Yes	Critically low
Young[45]	2007	Yes	No	Yes	No	No	Yes	Yes	Yes	No	No	Yes	No	No	Yes	Yes	No	Critically low
Wouters[44]	2012	Yes	No	Yes	No	Yes	Yes	Yes	Yes	Partial Yes	No	Yes	Yes	Yes	Yes	Yes	Yes	Critically low
Macedo[36]	2017	Yes	No	Yes	No	Yes	Yes	No	Partial Yes	Partial Yes	No	No	Yes	Yes	Yes	No	No	Critically low
Stengel[42]	2004	Yes	No	Yes	Partial Yes	No	Yes	No	Yes	Partial Yes	No	No	No	No	Yes	No	Yes	Critically low

Description of items:

item 1: Did the research questions and inclusion criteria for the review include the components of PICO?
item 2: Did the report of the review contain an explicit statement that review methods were established prior to the conduct of the review and did the report justify any significant deviations from the protocol?
item 3: Did the review authors explain their selection of the study designs for inclusion in the review?
item 4: Did the review authors use a comprehensive literature search strategy?
item 5: Did the review authors perform study selection in duplicate?
item 6: Did the review authors perform data extraction in duplicate?
item 7: Did the review authors provide a list of excluded studies and justify the exclusions?
item 8: Did the review authors describe the included studies in adequate detail?
item 9: Did the review authors use a satisfactory technique for assessing the risk of bias (RoB) in individual studies that were included in the review?
item 10: Did the review authors report on the sources of funding for the studies included in the review?
item 11: If meta-analysis was performed, did the review authors use appropriate methods for statistical combination of results?
item 12: If meta-analysis was performed, did the review authors assess the potential impact of RoB in individual studies on the results of the meta-analysis or other evidence synthesis?
item 13: Did the review authors account for RoB in individual studies when interpreting/discussing the results of the review?
item 14: Did the review authors provide a satisfactory explanation for, and discussion of, any heterogeneity observed in the results of the review?
item 15: If they performed quantitative synthesis, did the review authors carry out an adequate investigation of publication bias (small study bias) and discuss its likely impact on the results of the review?
item 16: Did the review authors report any potential sources of conflict of interest, including any funding they received for conducting the review?

Yellow items indicate critical domains, and white items indicate non-critical weaknesses in AMSTAR 2 assessment.

Ratings

- **High:** no or on non-critical weakness
- **Moderate:** more than one non-critical weakness
- **Low:** one critical flaw with or without non-critical weakness
- **Critically low:** more than one critical flaw with or without non-critical weakness

Author(s):
Date:
Question: High volume hosptal compared to low volume hospital for Hospital mortality
Setting:
Bibliography:

Certainty assessment							№ of patients		Effect		Certainty	Importance
№ of studies	Study design	Risk of bias	Inconsistency	Indirectness	Imprecision	Other considerations	high volume hospital	low volume hospital	Relative (95% CI)	Absolute (95% CI)		
mortality (Lung cancer resection)												
8	observational studies	serious	serious [a]	not serious	not serious	none			**OR 0.71** (0.62 to 0.81)	**1 fewer per 1,000** (from 1 fewer to 1 fewer)	⨁◯◯◯ VERY LOW	CRITICAL
mortality (Thyroidectomy)												
2	observational studies	serious	serious [a]	not serious	not serious	strong association	5/63003 (0.0%)	18/62799 (0.0%)	**OR 0.26** (0.05 to 1.37)	**0 fewer per 1,000** (from 0 fewer to 0 fewer)	⨁◯◯◯ VERY LOW	CRITICAL
mortality (Repair of ruptured intracranial aneurysm)												
4	observational studies	serious	very serious [b,c]	not serious	not serious	none	2883/17767 (16.2%)	2521/13863 (18.2%)	**OR 0.77** (0.60 to 0.97)	**36 fewer per 1,000** (from 4 fewer to 64 fewer)	⨁◯◯◯ VERY LOW	CRITICAL
Mortality (Bariatric surgery)												
7	observational studies	serious	very serious [b]	not serious	not serious	strong association	308/127991 (0.2%)	2773/127339 (2.2%)	**OR 0.26** (0.10 to 0.66)	**16 fewer per 1,000** (from 7 fewer to 20 fewer)	⨁◯◯◯ VERY LOW	CRITICAL
mortality (Esophageal resection)												
9	observational studies	serious	very serious	not serious	not serious	strong association	443/15713 (2.8%)	1028/12130 (8.5%)	**OR 0.29** (0.16 to 0.53)	**59 fewer per 1,000** (from 38 fewer to 70 fewer)	⨁◯◯◯ VERY LOW	CRITICAL
mortality (Pancreaticoduodenectomy)												

12	observational studies	serious	very serious	not serious	not serious	strong association dose response gradient	523/20440 (2.6%)	2146/36213 (5.9%)	**OR 0.42** (0.35 to 0.51)	**33 fewer per 1,000** (from 28 fewer to 38 fewer)	⨁◯◯◯ VERY LOW	CRITICAL
mortality (Breast cancer)												
3	observational studies	serious	very serious	not serious	not serious	strong association	44/80768 (0.1%)	110/74347 (0.1%)	**OR 0.29** (0.11 to 0.74)	**1 fewer per 1,000** (from 0 fewer to 1 fewer)	⨁◯◯◯ VERY LOW	CRITICAL
mortality (Cystectomy)												
7	observational studies	serious	serious	not serious	not serious	none			**OR 0.56** (0.43 to 0.72)	**1 fewer per 1,000** (from 0 fewer to 1 fewer)	⨁◯◯◯ VERY LOW	CRITICAL
mortality (Repair of elective AAA)												
23	observational studies	serious	not serious	not serious	not serious	dose response gradient			**OR 0.67** (0.64 to 0.72)	**1 fewer per 1,000** (from 1 fewer to 1 fewer)	⨁⨁◯◯ LOW	
mortality (Repair of ruptured AAA)												
8	observational studies	serious	serious [a]	not serious	not serious	dose response gradient			**OR 0.70** (0.61 to 0.81)	**1 fewer per 1,000** (from 1 fewer to 1 fewer)	⨁◯◯◯ VERY LOW	CRITICAL
mortality (Liver cancer)												
12	observational studies	serious	very serious	not serious	not serious	strong association	853/29442 (2.9%)	1283/25993 (4.9%)	**OR 0.49** (0.39 to 0.61)	**25 fewer per 1,000** (from 19 fewer to 30 fewer)	⨁◯◯◯ VERY LOW	CRITICAL
mortality (Lower limb arterial surgery)												
4	observational studies	serious	very serious	not serious	not serious	publication bias strongly suspected	9229/182437 (5.1%)	7280/128800 (5.7%)	**OR 0.81** (0.72 to 0.91)	**10 fewer per 1,000** (from 5 fewer to 15 fewer)	⨁◯◯◯ VERY LOW	CRITICAL
mortality (Carotid endarterectomy)												

11	observational studies	serious	serious [a]	not serious	not serious	none			**OR 0.79** (0.71 to 0.88)	**1 fewer per 1,000** (from 1 fewer to 1 fewer)	⨁◯◯◯ VERY LOW	CRITICAL
mortality (CABG)												
6	observational studies	serious	very serious	not serious	not serious	none	4086/121292 (3.4%)	1774/32392 (5.5%)	**OR 0.68** (0.57 to 0.81)	**17 fewer per 1,000** (from 10 fewer to 23 fewer)	⨁◯◯◯ VERY LOW	CRITICAL
mortality (Colorectal cancer)												
7	observational studies	not serious	very serious	not serious	not serious	publication bias strongly suspected	2677/56792 (4.7%)	2887/57003 (5.1%)	**OR 0.75** (0.55 to 1.00)	**12 fewer per 1,000** (from 0 fewer to 22 fewer)	⨁◯◯◯ VERY LOW	CRITICAL
mortality (Colon cancer)												
14	observational studies	not serious	very serious	not serious	not serious	none	6216/154543 (4.0%)	8433/155150 (5.4%)	**OR 0.75** (0.67 to 0.83)	**13 fewer per 1,000** (from 9 fewer to 17 fewer)	⨁◯◯◯ VERY LOW	CRITICAL
mortality (Rectal cancer)												
7	observational studies	not serious	very serious	not serious	not serious	none	478/12302 (3.9%)	309/6081 (5.1%)	**OR 0.75** (0.54 to 1.05)	**12 fewer per 1,000** (from 2 more to 23 fewer)	⨁◯◯◯ VERY LOW	CRITICAL
mortality (Total knee replacement)												
4	observational studies	serious	not serious [b]	not serious	not serious	dose response gradient			**OR 0.73** (0.61 to 0.88)	**1 fewer per 1,000** (from 1 fewer to 1 fewer)	⨁⨁◯◯ LOW	CRITICAL
mortality (Nephrectomy)												

14	observational studies	serious	serious	very serious [b]	not serious	none	1333/92175 (1.4%)	1980/119669 (1.7%)	**OR 0.73** (0.61 to 0.88)	**4 fewer per 1,000** (from 2 fewer to 6 fewer)	⨁◯◯◯ VERY LOW	CRITICAL

CI: Confidence interval; **OR:** Odds ratio

Explanations

a. I square 40-60
b. I square > 60
c. I square < 40

Author(s):
Date:
Question: High surgeon volume compared to Low surgeon volume for mortality
Setting:
Bibliography:

Certainty assessment							№ of patients		Effect		Certainty	Importance
**№ of studies**	**Study design**	**Risk of bias**	**Inconsistency**	**Indirectness**	**Imprecision**	**Other considerations**	**High surgeon volume**	**Low surgeon volume**	**Relative (95% CI)**	**Absolute (95% CI)**		
Mortality (Gynecogical oncology)												
4	observational studies	serious	not serious	not serious	not serious	none	51/6849 (0.7%)	95/7791 (1.2%)	**OR 0.52** (0.35 to 0.77)	**6 fewer per 1,000** (from 3 fewer to 8 fewer)	⨁◯◯◯ VERY LOW	CRITICAL
Mortality (Lung cancer resection)												
2	observational studies	serious	very serious	not serious	not serious	strong association	413/9703 (4.3%)	505/9273 (5.4%)	**OR 0.48** (0.16 to 1.47)	**28 fewer per 1,000** (from 24 more to 45 fewer)	⨁◯◯◯ VERY LOW	CRITICAL
Mortality (thyroidectomy)												
3	observational studies	serious	very serious	not serious	not serious	very strong association	16/78286 (0.0%)	223/88607 (0.3%)	**OR 0.18** (0.06 to 0.58)	**2 fewer per 1,000** (from 1 fewer to 2 fewer)	⨁◯◯◯ VERY LOW	CRITICAL
Mortality (Bariatric surgery)												
4	observational studies	serious	serious	not serious	not serious	publication bias strongly suspected [a]	66/16100 (0.4%)	130/4700 (2.8%)	**OR 0.20** (0.09 to 0.46)	**22 fewer per 1,000** (from 15 fewer to 25 fewer)	⨁◯◯◯ VERY LOW	CRITICAL
Mortality (Radical cystectomy)												
2	observational studies	serious	not serious	not serious	not serious	dose response gradient	125/5267 (2.4%)	234/5430 (4.3%)	**OR 0.56** (0.45 to 0.70)	**18 fewer per 1,000** (from 13 fewer to 23 fewer)	⨁⨁◯◯ LOW	CRITICAL
Mortality (Abdominal aortic aneurysm)												
6	observational studies	serious	serious [b]	not serious	not serious	dose response gradient			**OR 0.57** (0.48 to 0.67)	**1 fewer per 1,000** (from 0 fewer to 1 fewer)	⨁◯◯◯ VERY LOW	CRITICAL

Mortality (Colorectal cancer)												
5	observational studies	not serious	not serious	not serious	not serious	none	416/12268 (3.4%)	515/11376 (4.5%)	**OR 0.65** (0.56 to 76.00)	**15 fewer per 1,000** (from 19 fewer to 738 more)	⊕⊕◯◯ LOW	CRITICAL
Mortality (Colon cancer)												
7	observational studies	not serious	very serious	not serious	not serious	none	2395/76239 (3.1%)	3314/75992 (4.4%)	**OR 0.63** (0.51 to 0.76)	**16 fewer per 1,000** (from 10 fewer to 21 fewer)	⊕◯◯◯ VERY LOW	CRITICAL
Mortality (Rectal cancer)												
4	observational studies	not serious	not serious	not serious	not serious	none	121/2795 (4.3%)	174/2945 (5.9%)	**OR 0.73** (0.54 to 0.98)	**15 fewer per 1,000** (from 1 fewer to 26 fewer)	⊕⊕◯◯ LOW	CRITICAL
Mortality (Oesophageal cancer)												
4	observational studies	serious	very serious	not serious	not serious	publication bias strongly suspected			**OR 0.65** (0.36 to 1.14)	**1 fewer per 1,000** (from 0 fewer to 1 fewer)	⊕◯◯◯ VERY LOW	CRITICAL
Mortality (Pancreaticoduodenectomy)												
10	observational studies	serious	not serious	not serious	not serious	strong association	281/11780 (2.4%)	1417/22680 (6.2%)	**OR 0.38** (0.30 to 0.49)	**38 fewer per 1,000** (from 31 fewer to 43 fewer)	⊕⊕◯◯ LOW	CRITICAL

CI: Confidence interval; **OR:** Odds ratio

Explanations

a. Egger's P value < 0.1
b. I square > 60

Supplemental Content 5. Sensitivity analysis on two different meta-analyses for the association between hospital volume and mortality

Type of surgery	First author (Year of publication)	Number of studies	Number of cases	Number of patients	Primary outcome (mortality)	High-volume threshold per year	Summary odds ratio (95% CI)			Fixed *P* value	Random *P* value‡	Heterogeneity		Egger's *P* value	Excess significance				95% Prediction interval	Evidence class	AMSTAR 2	GRADE
							Fixed effects OR	Random effects OR	Largest study OR			I^2 (%)	P (Q test)		Observed	Expected	*P* value (chi)	*P* value (bin)				
**Esophageal resection**	Markar, 2012[37]	9	1471	27843	In-hospital or 30-day	9-346	0.29 (0.26-0.33)	0.29 (0.16-0.53)	0.41 (0.33-0.52)	p<0.001	9×10^{-5}	95	P<0.001	0.71	6	7.45	0.2	0.19	0.04-2.40	Non-significant	Critically low	Very low
**Esophageal resection**	Wouters, 2012[44]	16	(NA)	(NA)	Postoperative	2-86	0.50 (0.45 to 0.55)	0.44 (0.36 to 0.53)	0.62 (0.53 to 0.73)	p<0.001	4×10^{-16}	60	0.001	0.027	(NA)	(NA)	(NA)	(NA)	0.23-0.83	IV	Critically low	Very low

CI=confidence interval; NA=not applicable; AMSTAR=A MeaSurement Tool to Assess systematic Reviews; GRADE=Grading of Recommendations, Assessment, Development and Evaluation.
Excess significance cannot be calculated when 2×2 contingency tables were not available.
‡ For use of stratification of evidence, only random-effects P values are expressed as an exponential form when applicable.
